# Glutathione-induced drought stress tolerance in mung bean: coordinated roles of the antioxidant defence and methylglyoxal detoxification systems

**DOI:** 10.1093/aobpla/plv069

**Published:** 2015-07-01

**Authors:** Kamrun Nahar, Mirza Hasanuzzaman, Md. Mahabub Alam, Masayuki Fujita

**Affiliations:** 1 Laboratory of Plant Stress Responses, Department of Applied Biological Science, Faculty of Agriculture, Kagawa University, Miki-cho, Kita-gun, Kagawa 761-0795, Japan; 2 Department of Agricultural Botany, Faculty of Agriculture, Sher-e-Bangla Agricultural University, Sher-e-Bangla Nagar, Dhaka 1207, Bangladesh; 3 Department of Agronomy, Faculty of Agriculture, Sher-e-Bangla Agricultural University, Sher-e-Bangla Nagar, Dhaka 1207, Bangladesh

**Keywords:** Abiotic stress tolerance, antioxidant defence, drought, glutathione, methylglyoxal, reactive oxygen species

## Abstract

Drought is considered one of the most acute environmental stresses presently affecting agriculture. We studied the role of exogenous glutathione (GSH) in conferring drought stress tolerance in mung bean (*Vigna radiata* L. cv. Binamoog-1) seedlings by examining the antioxidant defence and methylglyoxal (MG) detoxification systems and physiological features. Six-day-old seedlings were exposed to drought stress (−0.7 MPa), induced by polyethylene glycol alone and in combination with GSH (1 mM) for 24 and 48 h. Drought stress decreased seedling dry weight and leaf area; resulted in oxidative stress as evidenced by histochemical detection of hydrogen peroxide (H_2_O_2_) and ${{\text{O}}_{2}}^{\cdot -}$ in the leaves; increased lipid peroxidation (malondialdehyde), reactive oxygen species like H_2_O_2_ content and ${{\text{O}}_{2}}^{\cdot -}$ generation rate and lipoxygenase activity; and increased the MG level. Drought decreased leaf succulence, leaf chlorophyll and relative water content (RWC); increased proline (Pro); decreased ascorbate (AsA); increased endogenous GSH and glutathione disulfide (GSSG) content; decreased the GSH/GSSG ratio; increased ascorbate peroxidase and glutathione *S*-transferase activities; and decreased the activities of monodehydroascorbate reductase (MDHAR), dehydroascorbate reductase (DHAR) and catalase. The activities of glyoxalase I (Gly I) and glyoxalase II (Gly II) increased due to drought stress. In contrast to drought stress alone, exogenous GSH enhanced most of the components of the antioxidant and glyoxalase systems in drought-affected mung bean seedlings at 24 h, but GSH did not significantly affect AsA, Pro, RWC, leaf succulence and the activities of Gly I and DHAR after 48 h of stress. Thus, exogenous GSH supplementation with drought significantly enhanced the antioxidant components and successively reduced oxidative damage, and GSH up-regulated the glyoxalase system and reduced MG toxicity, which played a significant role in improving the physiological features and drought tolerance.

## Abstract (precis)

Drought is considered one of the most acute abiotic stresses presently affecting agriculture. Plant tolerance to drought stress often depends on their tolerance against oxidative stress, which is acquired largely by strong antioxidant defense. In our paper we studied the glutathione-induced drought stress tolerance in *Vigna radiata* seedlings. Drought decreased growth, resulted in oxidative stress and increased methylglyoxal toxicity. But exogenous GSH enhanced components of the antioxidant system in drought-affected mung bean seedlings, which alleviated oxidative damage, up-regulated the glyoxalase system, reduced MG toxicity, and modulated the proline and water content, contributing to drought tolerance.

## Introduction

With increasing population and economic development, the agricultural, energy and industrial sectors of the world's economy have expanded, resulting in a manifold increase in demand for water. Anthropogenic activities have greatly increased environmental pollution, which limits the availability of fresh or quality water. Climate change has also contributed to water scarcity. As a result of all these factors, in recent decades droughts have occurred frequently and with higher severity (Ceccarelli *et al*. 2010; Mishra and Singh 2010).

Drought is recognized as one of the most complex and devastating threats to plants because of its multiple damaging effects. In plants, the effects of drought damage begin primarily with disruption of osmotic balance, which gradually develops into metabolic and physiological disorders (Pennisi 2008). As a result, drought can reduce stomatal conductance; impair and reduce the membrane electron transport rate, CO_2_ diffusion, carboxylation efficiency, water-use efficiency, respiration, transpiration and photosynthesis; damage organelle membranes; limit growth and developmental processes; and reduce yields. These damaging effects are very common under drought stress (Lawlor 2002; Pinheiro and Chaves 2011). The severity of drought stress is increasing day by day and it has been estimated that it will cause losses in global crop production of up to 30 % by 2025, compared with current yields (Zhang 2011). Under drought stress, constraints in stomatal movement, photosynthetic reaction centre, biochemical reactions of the Calvin cycle, electron transport system or enzyme activities during photosynthesis may result in overproduction of reactive oxygen species (ROS) like ${{\text{O}}_{2}}^{\cdot -},$ ^1^O_2_, hydrogen peroxide (H_2_O_2_) and OH^·^, which cause oxidative stress and damage cells or their components by disrupting the enzymatic functions of biochemical reactions (Faize *et al*. 2011; Sorkheh *et al*. 2011). Methylglyoxal (MG) is a cytotoxic compound generated through the glycolysis pathway of eukaryotic cells (Yadav *et al*. 2005*a*). The activity of MG synthase may lead to overproduction of MG (Martins *et al*. 2001), which can be generated through fatty acid or aminoacetone metabolism (Casazza *et al*. 1984). The small amount of MG produced under normal growing conditions can be easily and readily scavenged by a plant's glyoxalase system. However, stress conditions result in higher amounts of MG production and accumulation. Depending upon plant species, MG concentration increases 2- to 6-fold in response to different stresses such as salinity, drought and cold (Yadav *et al*. 2005*a*, *b*, *c*), and it can create similar oxidative stress effects on plant cells like increased ROS (Wang *et al*. 2009; Desai *et al*. 2010).

Plant tolerance to abiotic stress largely depends on their tolerance against oxidative stress or MG stress, which is acquired largely by strong antioxidant and glyoxalase systems (Mittler *et al*. 2004; Gill and Tuteja 2010; Hasanuzzaman and Fujita 2011; Hasanuzzaman *et al*. 2011). The antioxidant system through step-by-step reactions scavenges ROS by means of non-enzymatic antioxidants (ascorbic acid (AsA); glutathione (GSH); phenolic compounds, alkaloids, non-protein amino acids and α-tocopherols) together with antioxidant enzymes (superoxide dismutase (SOD); catalase (CAT); ascorbate peroxidase (APX); glutathione reductase (GR); monodehydroascorbate reductase (MDHAR); dehydroascorbate reductase (DHAR); glutathione peroxidase (GPX); and glutathione *S*-transferase (GST)) (Pang and Wang 2008; Gill and Tuteja 2010; Hasanuzzaman *et al*. 2012). On the other hand, MG is detoxified in the glyoxalase system where the enzymes glyoxalase I (Gly I) and glyoxalase II (Gly II) detoxify MG using GSH as a substrate. Therefore, effective ROS or MG detoxification indicates an appropriately functioning antioxidant or glyoxalase system (Yadav *et al*. 2005*a*; Gill and Tuteja 2010; Hasanuzzaman *et al*. 2011, 2012). Endogenous GSH plays an important role in the antioxidant and glyoxalase systems that are vital for ROS and MG detoxification, respectively. Glutathione has diversified properties that are required for a broad range of biochemical functions. Present in cytosol and in almost all cell organelles (including chloroplasts, endoplasmic reticula, vacuoles and mitochondria), GSH performs and accelerates various biochemical functions (Foyer and Noctor 2003). Glutathione acts as a substrate or co-factor for a number of biochemical reactions; it interacts with hormones and redox molecules, and participates in stress-induced signal transduction (Foyer and Noctor 2005*a*, *b*; Szalai *et al*. 2009). Glutathione is well recognized in enhancing stress and disease resistance in humans and animals (Franco *et al*. 2014; Ghosh *et al*. 2014; Nadeem *et al*. 2014). However, there are very few reports regarding the effects of exogenously applied GSH on plants, especially under abiotic stress (Kattab 2007; Salama and Al-Mutawa 2009; Wang *et al*. 2011; Nahar *et al*. 2015*a*, *b*). Many aspects of the role of GSH in plants under abiotic stress remain unknown. Considering these aspects, the present study looks at how exogenous GSH application in mung bean seedlings can confer drought tolerance. In particular, we examined the antioxidant system and oxidative stress, glyoxalase system and MG level and some other physiological features of mung bean seedlings. To the best of our knowledge, this is the first study on the regulatory role of exogenous GSH on the antioxidant and glyoxalase systems in mung bean plants under drought stress.

## Methods

### Plant materials and stress treatments

Mung bean (*Vigna radiata* L. cv. Binamoog-1) seeds were sown in petri dishes and placed in the dark for 48 h for germination. One petri dish contained 30 germinated seedlings and was considered as one set of seedlings. Germinated seedlings were grown under controlled conditions (light, 350 μmol photon m^−2^ s^−1^; temperature, 25 ± 2 °C; relative humidity, 65–70 %) in a growth chamber, with 10 000-fold diluted Hyponex solution (Hyponex, Japan) applied as nutrient. Six-day-old seedlings (two sets) were exposed to drought stress (−0.7 MPa by treating with Hyponex solution containing 25 % polyethylene glycol 6000, PEG). Another two sets of seedlings were supplemented with 1 mM GSH with drought stress. A further two sets of seedlings were grown with 1 mM GSH in the Hyponex solution. Control seedlings were grown with Hyponex solution only. The experiment was repeated three times. Data were taken after 24 and 48 h. The experiment was conducted with a completely randomized design with three replications.

### Determination of growth parameters

Ten randomly selected fresh seedlings from each treatment were dried at 80 °C for 48 h, then weighed and considered as dry weight (DW), expressed in g. Leaf area (*A*) was determined and expressed in cm^2^.

### Measurement of leaf relative water content and leaf succulence

Leaf relative water content (RWC) was measured according to Barrs and Weatherley (1962). Fresh weight (FW), turgid weight (TW) and DW of leaves were measured, and leaf RWC was calculated using the following formula: leaf RWC (%) = [(FW − DW)/(TW − DW)] × 100. Leaf succulence was determined according to Silveira *et al*. (2009) using the following equation: leaf succulence = FW/*A* (where *A* is leaf area) and expressed as mg FW cm^−2^.

### Measurement of chlorophyll content

Leaves were extracted with 80 % v/v acetone (centrifuging at 5000 × *g*), absorbance of the supernatant of the plant samples was measured at 663 and 645 nm and chlorophyll (chl) content was calculated according to Arnon (1949).

### Measurement of proline content

Proline (Pro) was appraised according to Bates *et al*. (1973). Leaves were homogenized in 3 % sulfosalicylic acid and centrifuged at 11 500 × *g*. The supernatant was mixed with acid ninhydrin with glacial acetic acid and phosphoric acid. After incubating the mixture at 100 °C for 1 h and cooling, toluene was added, and after several minutes a chromophore containing toluene was read spectrophotometrically at 520 nm.

### Histochemical localization of hydrogen peroxide and O_2_^·−^

Hydrogen peroxide and ${{\text{O}}_{2}}^{\cdot -}$ were localized histochemically (Chen *et al*. 2010) by staining leaves with 1 % 3,3-diaminobenzidine (DAB) and 0.1 % nitroblue tetrazolium (NBT) solution, respectively. Leaves were collected from the same position in plants of the same age, and were then immersed in those solutions until brown spots appeared due to the reaction of DAB with H_2_O_2_ or dark blue spots appeared due to the reaction of NBT with ${{\text{O}}_{2}}^{\cdot -}.$ Controls (drought treatment) were performed with 10 mM ascorbic acid (removing the H_2_O_2_) or 10 mM MnCl_2_ (${{\text{O}}_{2}}^{\cdot -}$ removing reagent) (Ikbal *et al*. 2014). All leaf samples from the different treatments were incubated for the same duration. After that, the leaves were drenched in boiling ethanol to see the spots.

### Determination of lipid peroxidation

The level of lipid peroxidation was measured by estimating malondialdehyde (MDA, a product of lipid peroxidation), using thiobarbituric acid (TBA) according to Heath and Packer (1968) with modifications (Hasanuzzaman *et al*. 2011).

### Measurement of hydrogen peroxide content

Hydrogen peroxide was assayed according to Yu *et al*. (2003) by extracting leaves in potassium phosphate (K-P) buffer (pH 6.5) (centrifuging at 11 500 × *g*), then mixing with TiCl_4_ in 20 % H_2_SO_4_ (v/v). Absorbance was measured spectrophotometrically at 410 nm.

### Measurement of O_2_^·−^ generation rate

The rate of ${{\text{O}}_{2}}^{\cdot -}$ generation was determined following Yang *et al*. (2011) with some modifications. Fresh leaves were homogenized in 65 mM K-P buffer solution (pH 7.8) and centrifuged at 5000 × *g*. Supernatant was mixed with extraction buffer and 10 mM hydroxylamine hydrochloride and incubated at 25 °C for 20 min. Then, 17 mM sulfanilamide and 7 mM naphthylamine were added and the mixture was incubated again at 25 °C for 20 min. Absorbance was measured at 530 nm. The rate of ${{\text{O}}_{2}}^{\cdot -}$ generation was calculated by using the standard curve of NaNO_2_ (Elstner and Heupel 1976).

### Measurement of methylglyoxal level

Leaves were homogenized in 5 % perchloric acid and centrifuged at 4 °C for 10 min at 11 000 × *g*. The supernatant was decolourized by charcoal and neutralized by saturated potassium carbonate solution. The neutralized supernatant was used for MG estimation by adding sodium dihydrogen phosphate and *n*-acetyl-l-cysteine to a final volume of 1 mL. Formation of the product *N*-*α*-acetyl-*S*-(1-hydroxy-2-oxo-prop-1-yl)cysteine was recorded after 10 min at 288 nm (Wild *et al*. 2012). The MG content was calculated by using a standard curve of known concentration of MG and expressed as µmol g^−1^ FW.

### Extraction and measurement of ascorbate and glutathione

Leaves (0.5 g) were homogenized in 5 % meta-phosphoric acid containing 1 mM EDTA, centrifuged at 11 500 × *g* for 15 min at 4 °C and the supernatant was collected for analysis of AsA and GSH. Ascorbate content was determined following the method of Huang *et al*. (2005) with some modifications (Hasanuzzaman *et al*. 2011). The glutathione pool was assayed according to Yu *et al*. (2003) with modifications (Paradiso *et al*. 2008; Hasanuzzaman *et al*. 2011). Standard curves with known concentrations of AsA, GSH and glutathione disulfide (GSSG) were used to determine their contents in the plant samples.

### Protein determination

The protein concentration of each sample was determined following the method of Bradford (1976) using BSA as a protein standard.

### Enzyme extraction and assays

Leaves were homogenized with 50 mM K-P buffer (pH 7.0) containing 100 mM KCl, 1 mM AsA, 5 mM β-mercaptoethanol and 10 % (w/v) glycerol in pre-chilled mortars and pestles. Homogenates were centrifuged at 11 500 × *g* and supernatants were used for enzyme activity assay.

Ascorbate peroxidase (EC: 1.11.1.11) activity (Nakano and Asada 1981) assay: reaction buffer solution contained 50 mM K-P buffer (pH 7.0), 0.5 mM AsA, 0.1 mM H_2_O_2_, 0.1 mM EDTA and enzyme extract (final volume 700 μL). The reaction was started by adding H_2_O_2_ and the decrease in the absorbance at 290 nm was recorded for 1 min. Monodehydroascorbate reductase (EC: 1.6.5.4) activity (Hossain *et al*. 1984): the reaction mixture contained 50 mM Tris–HCl buffer (pH 7.5), 0.2 mM NADPH, 2.5 mM AsA, 0.5 unit of AO and enzyme solution (final volume 700 μL). The reaction was started by adding AO to record absorbance at 340 nm.

Dehydroascorbate reductase (EC: 1.8.5.1) activity (Nakano and Asada 1981): the reaction buffer contained 50 mM K-P buffer (pH 7.0), 2.5 mM GSH and 0.1 mM DHA (dehydroascorbate). Activity was calculated from the change in absorbance at 265 nm for 1 min using an extinction coefficient of 14 mM^−1^ cm^−1^.

Glutathione reductase (EC: 1.6.4.2) activity (Hasanuzzaman *et al*. 2011): The reaction mixture contained 0.1 M K-P buffer (pH 7.0), 1 mM EDTA, 1 mM GSSG, 0.2 mM NADPH and enzyme solution (final volume 1 mL). The reaction was initiated with GSSG and the decrease in absorbance was recorded at 340 nm for 1 min.

Superoxide dismutase (EC 1.15.1.1) activity (El-Shabrawi *et al*. 2010): SOD activity was assayed using a xanthine–xanthine oxidase system. The reaction mixture contained K-P buffer (50 mM), NBT (2.24 mM), CAT (0.1 units), xanthine oxidase (0.1 units), xanthine (2.36 mM) and enzyme extract. Catalase was added to avoid H_2_O_2_-mediated inactivation of CuZn–SOD. The change in absorbance was read at 560 nm.

Catalase (EC: 1.11.1.6) activity (Hasanuzzaman *et al*. 2011): the decrease in absorbance (by decomposition of H_2_O_2_) was read at 240 nm for 1 min.

Glutathione *S*-transferase (EC: 2.5.1.18) activity (Hossain *et al*. 2006): the reaction mixture contained 100 mM Tris–HCl buffer (pH 6.5), 1.5 mM GSH, 1 mM 1-chloro-2,4-dinitrobenzene (CDNB) and enzyme solution (final volume 700 μL). The reaction was initiated by CDNB; the increase in absorbance was measured at 340 nm for 1 min.

Glutathione peroxidase (EC: 1.11.1.9) activity (Elia *et al*. 2003; Hasanuzzaman *et al*. 2011): The reaction mixture consisted of 100 mM K-P buffer (pH 7.0), 1 mM EDTA, 1 mM NaN_3_, 0.12 mM NADPH, 2 mM GSH, 1 unit GR, 0.6 mM H_2_O_2_ (as a substrate) and 20 μL enzyme. The oxidation of NADPH was read at 340 nm for 1 min.

Glyoxalase I (EC: 4.4.1.5) (Hasanuzzaman *et al*. 2011): the assay mixture contained 100 mM K-P buffer (pH 7.0), 15 mM magnesium sulfate, 1.7 mM GSH and 3.5 mM MG. The reaction was started by adding MG; the increase in absorbance was recorded at 240 nm for 1 min.

Glyoxalase II (EC: 3.1.2.6) (Principato *et al*. 1987): the reaction mixture contained 100 mM Tris–HCl buffer (pH 7.2), 0.2 mM DTNB and 1 mM *S*-d-lactoylglutathione (SLG). The reaction was started by adding SLG and absorbance at 412 nm was monitored; activity was calculated using the extinction coefficient 13.6 mM^−1^ cm^−1^.

Lipoxygenase (LOX) (EC 1.13.11.12) activity (Doderer *et al*. 1992): LOX activity was assayed by recording the increase in absorbance at 234 nm using linoleic acid as a substrate. The activity was calculated using the extinction coefficient 25 mM^−1^ cm^−1^.

### Statistical analysis

The experiment was conducted using a completely randomized design, with three replications (i.e. the experiment was repeated three times under the same conditions). All data obtained were evaluated using multi-factor analysis of variance, with treatment (control, GSH, drought and drought + GSH), and time (24 and 48 h) as the grouping factors. If the treatment or treatment × time interaction were significant, we then compared all means using Duncan's multiple range tests in MSTAT-C software (Freed 1994).

## Results

### Oxidative stress indicators

**Figure 1.**
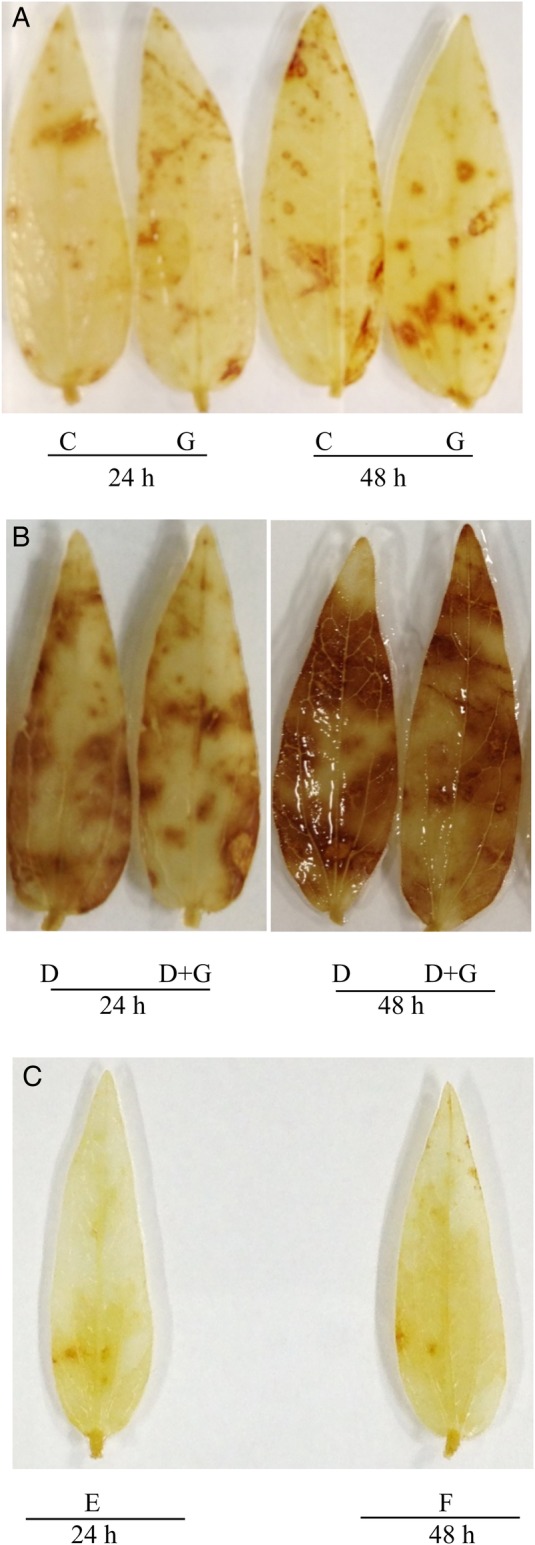
(A and B) Histochemical localization of H_2_O_2_ in leaves of mung bean seedlings. Here C, G, D and D + G indicate control, exogenous glutathione (GSH, 1 mM), drought stress (−0.7 MPa) and drought stress (−0.7 MPa) + exogenous glutathione (GSH, 1 mM), respectively. (C) Histochemical localization of H_2_O_2_ in leaves of drought-treated plants stained in 10 mM ascorbate where E and F indicate 24 and 48 h drought (control) treatment, respectively.

**Figure 2.**
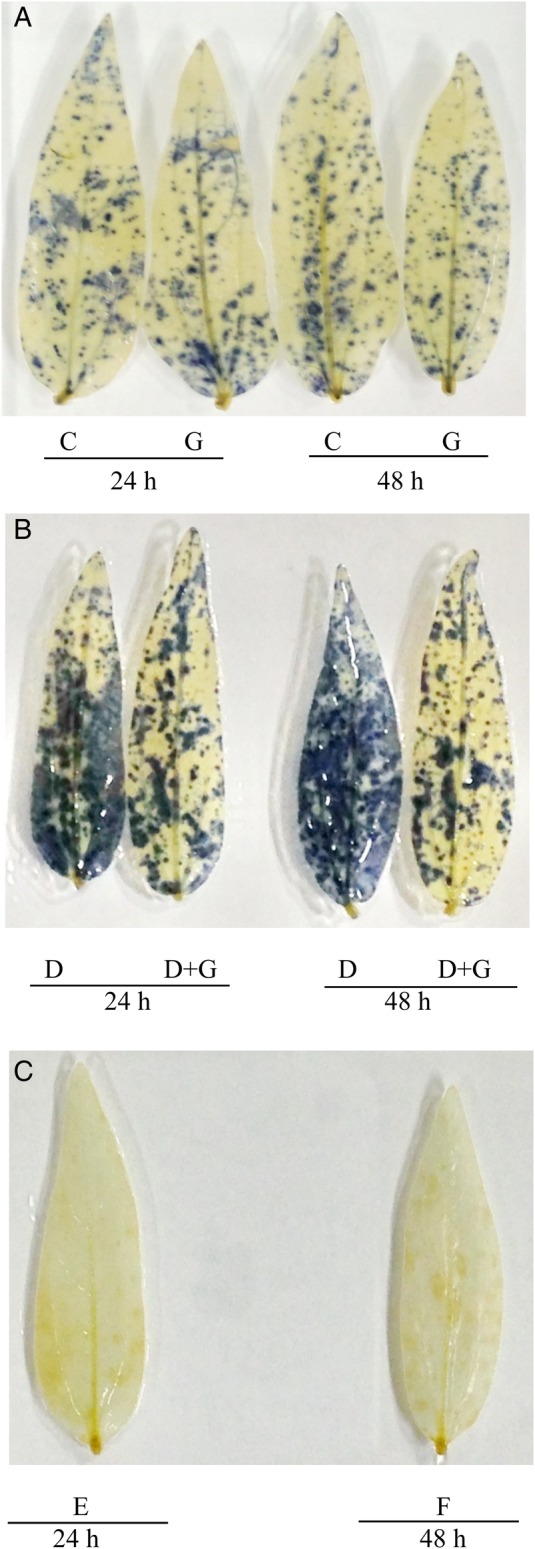
(A and B) Histochemical localization of ${{\text{O}}_{2}}^{\cdot -}$ in leaves of mung bean seedlings. Here C, G, D and D + G indicate control, exogenous glutathione (GSH, 1 mM), drought stress (−0.7 MPa) and drought stress (−0.7 MPa) + exogenous glutathione (GSH, 1 mM), respectively. (C) Histochemical localization of ${{\text{O}}_{2}}^{\cdot -}$ in leaves of drought-treated plants stained in 10 mM MnCl_2_ where E and F indicate 24 and 48 h drought (control) treatment, respectively.

Histochemical staining was performed to localize H_2_O_2_ and ${{\text{O}}_{2}}^{\cdot -}$ in the leaves of the mung bean seedlings (Figs 1 and 2). A significant increase in the accumulation of H_2_O_2_, indicated by brown spots (Fig. 1B), and accumulation of ${{\text{O}}_{2}}^{\cdot -}$, indicated by dark blue spots (Fig. 2B), were noticed in the leaves of the mung bean seedlings under drought stress, and more spots on the leaves were evident after 48 h. Incubated leaves in 10 mM ascorbate or 10 mM MnCl_2_ removed the H_2_O_2_ or ${{\text{O}}_{2}}^{\cdot -}$ staining, respectively (Figs 1C and 2C), which indicated the specificity of staining. Hydrogen peroxide was suppressed by adding 10 mm ascorbic acid. The production of ${{\text{O}}_{2}}^{\cdot -}$ was suppressed by adding 10 mm MnCl_2_, which is considered a removing agent of ${{\text{O}}_{2}}^{\cdot -}$ (Hernández *et al*. 2001). However, GSH addition with drought stress reduced the spots of ${{\text{O}}_{2}}^{\cdot -}$ from the leaves. But exogenous GSH addition could not reduce the spots of H_2_O_2_ from mung bean leaves.

As summarized in Table 1, at least two treatment effects are significantly different, so a multiple comparison was performed to find out which was superior. Treatment factor, time factor and their interaction (treatment × time) as oxidative stress parameters are significantly different (at a probability level of *P* ≤ 0.01). The effect of the treatments either individually or their interaction was significant for the oxidative stress parameters (Table 1). In the case of interaction, the MDA and H_2_O_2_ levels increased significantly in the mung bean seedlings exposed to drought stress (Table 1). Compared with the non-stressed control, the MDA level rose by 119 and 193 %, and the H_2_O_2_ level increased by 38 and 114 % after 24 and 48 h, respectively. In contrast, compared with drought stress alone, exogenous GSH application with drought stress significantly reduced MDA at 24 and 48 h. Exogenous GSH did not significantly reduce H_2_O_2_ content and its values were statistically similar in the GSH-supplemented drought treatment and drought only treatment. Drought stress gradually increased the ${{\text{O}}_{2}}^{\cdot -}$ generation rate in the mung bean seedlings from 24 to 48 h (compared with control), which was reversed by exogenous GSH treatment. The ${{\text{O}}_{2}}^{\cdot -}$ generation rate decreased by 29 and 27 % after 24 and 48 h, respectively, compared with drought stress alone (Table 1).

**Table 1.** Malondialdehyde (MDA) and H_2_O_2_ content, ${{\text{O}}_{2}}^{\cdot -}$ generation rate and LOX activity in mung bean seedlings induced by exogenous glutathione (GSH, 1 mM) under drought stress (−0.7 MPa). Mean (±SE) was calculated from three replicates for each treatment. Values in a column with different letters are significantly different at *P* ≤ 0.01 (**) applying Duncan's multiple range test (DMRT).

Source of variation	MDA content (nmol g^−1^ FW)	H_2_O_2_ content (nmol g^−1^ FW)	O_2_^·−^ generation rate (nmol min^−1^ g^−1^ FW)	LOX activity (µmol min^−1^ mg^−1^ protein)
Treatment				
Control	29.42 c	18.33 b	10.80 c	32.31 b
GSH	25.68 c	20.16 b	11.60 bc	27.44 b
Drought	75.07 a	32.07 a	20.18 a	46.71 a
Drought + GSH	47.52 b	31.14 a	14.52 b	33.44 b
Level of significance	**	**	**	**
SE	2.22	0.97	0.73	1.84
*F* ratio (d.f. = 3,14)	103.44	54.65	33.80	20.13
Time				
24 h	39.73 b	23.70 b	13.21 b	35.39 a
48 h	49.11 a	27.15 a	15.34 a	34.55 b
Level of significance	**	**	**	**
SE	1.57	0.69	0.52	1.30
*F* ratio (d.f. = 1,14)	17.90	12.56	8.45	0.21
Treatment × time				
Control × 24 h	29.93 c	18.83 e	10.81 d	31.62 c
Control × 48 h	28.90 c	17.84 e	10.78 d	32.99 bc
GSH × 24 h	24.97 c	20.88 de	10.72 d	27.14 c
GSH × 48 h	26.38 c	19.43 e	12.48 cd	27.74 c
Drought × 24 h	65.44 b	25.90 cd	18.30 ab	49.01 a
Drought × 48 h	84.71 a	38.23 a	22.06 a	44.40 ab
Drought + GSH × 24 h	33.58 c	29.19 bc	13.02 cd	33.79 bc
Drought + GSH × 48 h	56.45 b	33.09 ab	16.02 bc	33.09 bc
Level of significance	**	**	**	**
SE	3.14	1.37	1.03	2.60
*F* ratio (d.f. = 3,14)	5.79	10.83	1.28	0.52

### Lipoxygenase activity

The treatment × time interaction was significant for LOX activity, which increased because of drought stress by 55 and 35 % after 24 and 48 h, respectively, compared with control. On the other hand, exogenous GSH application significantly reduced LOX activity in the drought-stressed mung bean seedlings after 24 h, compared with the activity in the drought-affected seedlings without GSH application. The decreased LOX activity in GSH-added drought treatment after 48 h was not statistically significant, compared with drought stress alone (Table 1).

### Growth parameters

Drought stress significantly reduced DW plant^−1^ by 14 % after 24 h and a higher reduction in DW plant^−1^ (by 22 %) was recorded after 48 h of drought stress, compared with the control seedlings. Drought stress also reduced leaf area by 20 and 29 % after 24 and 48 h, respectively, compared with the control. Later on, O_2_^·−^ seedling DW increased slightly in the plants with GSH supplementation which was not significant statistically. The increase in leaf area after GSH addition was statistically significant after 24 h (Table 2).

**Table 2.** Dry weight, leaf area, leaf RWC, leaf succulence, Pro content, chl *a*, chl *b* and total chl contents in mung bean seedlings induced by exogenous glutathione (GSH, 1 mM) under drought stress (−0.7 MPa). Mean (±SE) was calculated from three replicates for each treatment. Values in a column with different letters are significantly different at *P* ≤ 0.01 (**) or *P* ≤ 0.05 (*) applying the DMRT.

Source of variation	Dry weight (g seedling^−1^)	Leaf area (cm^2^)	Leaf RWC (%)	Leaf succulence (mg FW cm^−2^)	Pro content (µmol g^−1^ FW)	chl *a* (mg g^−1^ FW)	chl *b* (mg g^−1^ FW)	Total chl (mg g^−1^ FW)
Treatment								
Control	0.022 a	2.095 a	83.40 a	17.31 a	3.21 c	1.320 ab	1.34 a	2.69 a
GSH	0.023 a	2.06 a	82.94 a	17.93 a	4.37 c	1.355 a	1.38 a	2.68 a
Drought	0.018 b	1.58 b	62.49 b	15.24 b	10.49 b	1.103 c	1.14 b	2.24 b
Drought + GSH	0.02 ab	1.79 ab	69.38 b	16.53 a	13.51 a	1.257 b	1.39 a	2.65 a
Level of significance	*	*	**	*	**	**	**	**
SE	0.003	0.064	1.94	0.30	0.35	0.022	0.048	0.039
*F* ratio (d.f. = 3,14)	0.8	6.044	28.39	8.5	193.14	24.877	6.074	29.84
Time								
24 h	0.021 a	1.91 a	77.28 a	16.62 b	7.11 b	1.266 a	1.25 b	2.51 b
48 h	0.020 b	1.85 b	71.82 b	16.88 a	8.67 a	1.252 b	1.39 a	2.61 a
Level of significance	*	*	**	*	**	**	**	**
SE	0.002	0.019	1.37	0.66	0.25	0.016	0.035	0.028
*F* ratio (d.f. = 1,14)	0.8	1.2	7.91	35.4	19.38	0.40	7.67	5.84
Treatment × time								
Control × 24 h	0.022 a	2.07 a	84.60 a	17.44 a	3.45 d	1.310 a	1.23 ab	2.54 a
Control × 48 h	0.023 a	2.12 a	82.19 ab	17.19 a	2.96 d	1.330 a	1.46 a	2.79 a
GSH × 24 h	0.023 a	2.03 a	80.50 ab	18.16 a	4.49 d	1.347 a	1.33 ab	2.67 a
GSH × 48 h	0.023 a	2.09 a	85.37 a	17.70 a	4.24 d	1.363 a	1.44 a	2.68 a
Drought × 24 h	0.019 b	1.65 b	69.60 cd	14.51 b	8.83 c	1.127 bc	1.08 b	2.20 b
Drought × 48 h	0.018 b	1.51 b	55.37 e	15.98 b	18.49 a	1.080 c	1.19 ab	2.27 b
Drought + GSH × 24 h	0.020 ab	1.89 a	74.41 b	16.4 a	11.68 b	1.280 a	1.35 ab	2.62 a
Drought + GSH × 48 h	0.019 b	1.69 ab	64.34 de	16.67 ab	18.00 a	1.233 ab	1.44 a	2.67 a
Level of significance	*	*	**	*	**	**	**	**
SE	0.0045	0.015	2.74	1.12	0.50	0.032	0.068	0.055
*F* ratio (d.f. = 3,14)	0.9	1.3	4.74	8.79	9.94	0.707	0.413	1.904

### Leaf relative water content and leaf succulence

Drought stress significantly reduced leaf RWC in the mung bean seedlings by 18 and 33 % after 24 and 48 h, respectively, compared with the control. Exogenous application of GSH with drought significantly recovered leaf RWC after 24 h only (Table 2). Drought stress reduced leaf succulence significantly and it was restored by exogenous GSH after 24 h in the drought-affected plants (Table 2).

### Proline content

Proline content was significantly affected by the different treatments. Time or duration of drought stress also significantly affected the Pro content of the mung bean seedlings. The highest Pro level was observed in the GSH-supplemented drought treatment and the lowest Pro content was observed in the control and GSH-treated plants (which were not statistically different). On the other hand, higher Pro was observed after 48 h compared with 24 h. Interaction of treatment × time was also significant for Pro content. Drought markedly increased thr Pro content by 156 and 524 % after 24 and 48 h, respectively, compared with the non-stressed control treatment plants. Compared with drought stress alone, a further increase in Pro was observed with GSH supplementation with drought stress at 24 h, whereas 48 h GSH treatment with drought maintained statistically the same Pro level as in the drought treatment without GSH (Table 2).

### Chlorophyll content

Chlorophyll *a*, chl *b* and total chl (*a* + *b*) content are presented in Table 2 and their level of significance for treatment, time, and the interaction of treatment × time. Decreases in chl *a*, chl *b* and total chl content were the characteristic drought stress symptoms in the present study. For interaction of treatment × time, compared with the control, drought stress reduced chl *a* by 14 and 19 % at 24 and 48 h, respectively. Chlorophyll *b* content also decreased due to drought stress and as a result total chl also decreased (by 13 and 19 % after 24 and 48 h, respectively). Exogenous GSH supplementation with drought stress restored the reduced chl content in all the treated plants in this experiment except chl *b* at 48 h (Table 2).

### Ascorbate and glutathione levels

Non-enzymatic antioxidant components are presented in Table 3 where treatment, time and their interaction significantly affected (at *P* ≤ 0.01 probability level) these attributes. The AsA content in the control and GSH treatments was statistically similar at both 24 and 48 h. In the treatment × time interaction, drought stress reduced AsA content by 32 % after 24 h, compared with the non-stressed control seedlings. The decrease in AsA content at 48 h drought treatment was not so significant, compared with the 48 h control treatment. Exogenous GSH application with drought did not significantly increase the AsA level in the 24 h treatment (Table 3).

**Table 3.** Contents of AsA, GSH, GSSG and GSH/GSSG ratio in mung bean seedlings induced by exogenous glutathione (GSH, 1 mM) under drought stress (−0.7 MPa). Mean (±SE) was calculated from three replicates for each treatment. Values in a column with different letters are significantly different at *P* ≤ 0.01 (**) applying the DMRT.

Source of variation	AsA content (nmol g^−1^ FW)	GSH content (nmol g^−1^ FW)	GSSG content (nmol g^−1^ FW)	GSH/GSSG ratio
Treatment				
Control	2151.80 a	489.51 c	20.80 c	21.77 b
GSH	1893.98 ab	506.96 c	26.22 c	22.88 b
Drought	1552.75 b	899.65 b	68.45 a	14.67 c
Drought + GSH	1777.06 b	1217.33 a	39.44 b	30.92 a
Level of significance	**	**	**	**
SE	81.97	37.69	2.26	0.51
*F* ratio (d.f. = 3,14)	9.25	85.55	89.15	35.32
Time				
24 h	1949.03 a	709.05 b	32.63 b	23.52 a
48 h	1738.76 b	847.68 a	44.83 a	21.60 b
Level of significance	**	**	**	**
SE	57.96	26.65	1.60	0.79
*F* ratio (d.f. = 1,14)	6.58	13.53	29.23	2.94
Treatment × time				
Control × 24 h	2251.46 a	482.51 d	16.11 e	26.28 b
Control × 48 h	2052.14 ab	496.51 d	25.48 de	17.25 cd
GSH × 24 h	1959.63 abc	428.11 d	23.01 e	24.34 b
GSH × 48 h	1828.33 abc	585.80 d	29.42 cde	21.42 bc
Drought × 24 h	1529.91 bc	844.17 c	51.30 b	16.50 cd
Drought × 48 h	1579.59 bc	955.14 bc	85.61 a	12.83 d
Drought + GSH × 24 h	2055.12 ab	1081.38 b	40.08 bc	26.95 b
Drought + GSH × 48 h	1499.00 c	1353.27 a	38.80 bcd	34.89 a
Level of significance	**	**	**	**
SE	115.90	53.30	3.19	1.58
*F* ratio (d.f. = 3,14)	2.37	2.02	11.65	10.10

Different treatments significantly affected the endogenous GSH level. The GSH level was also significantly affected by time (duration of drought stress) and treatment × time interaction. Glutathione content increased markedly by 75 and 92 % after 24 and 48 h of drought stress, respectively, compared with the control seedlings. Endogenous GSH content increased again with exogenous GSH supplementation with drought stress (Table 3). Drought markedly increased the oxidized form of glutathione (GSSG) by 218 and 236 % after 24 and 48 h, respectively. Exogenous application of GSH significantly decreased the GSSG levels of the drought-affected seedlings after 48 h (Table 3). The GSH/GSSG ratio decreased in the drought stress-treated plants while supplementation of exogenous GSH with drought stress significantly restored and increased that ratio (Table 3).

### Antioxidant enzyme activity

Superoxide dismutase activity was significantly affected over time (significant at *P* ≤ 0.01), with an increase of 19 and 30 % after 24 and 48 h of drought stress, respectively. Exogenous GSH application with drought stress maintained the same SOD activity after 24 h and SOD increased by 19 % after 48 h, compared with drought stress alone (Table 4). A marked decline in CAT activity was observed in the drought-affected seedlings, compared with the non-stressed control seedlings, with a reduction of 25 and 28 % after 24 and 48 h, respectively. Adding GSH with drought did not significantly increase CAT activity further, compared with drought stress alone (Table 4). Drought stress and time (duration of drought exposure) significantly affected the activity of APX, MDHAR and DHAR, which were significant at the *P* ≤ 0.05 probability level. Compared with the control, drought stress increased APX activity by 21 % at both 24 and 48 h of drought stress. A further increase in APX activity was observed in the GSH-supplemented drought-treated seedlings at both 24 and 48 h, compared with the drought-treated seedlings alone (Table 4). Drought stress significantly reduced MDHAR activity and exogenous GSH supplementation with drought stress restored and increased MDHAR activity for both 24 and 48 h, compared with drought treatment alone (Table 4). Dehydroascorbate reductase activity was not affected significantly by drought, compared with the control. In contrast, GSH supplementation in the drought treatment enhanced DHAR activity by 68 % after 24 h, whereas GSH supplementation did not influence DHAR activity after 48 h (Table 4). Compared with the non-stressed control seedlings, GR activity was neither changed under drought stress nor affected by GSH supplementation with drought stress (Table 4). A significant effect of treatment and time on GST and GPX activity was observed. The interaction of treatment × time also significantly affected (at *P* ≤ 0.01 level) the activities of these enzymes. After 24 h of drought stress, GST activity increased significantly, compared with the control plants. Adding GSH with drought treatment maintained the same GST activity as with the single drought treatments after 24 h and its activity increased after 48 h (Table 4). Compared with the control seedlings, drought treatment did not alter GPX activity. However, GPX activity increased by 36 % with GSH-added drought treatment (after both 24 and 48 h), compared with drought treatment alone (Table 4).

**Table 4.** Activities of antioxidant enzymes; SOD, CAT, APX, MDHAR, DHAR and GR, GST and GPX in mung bean seedlings induced by exogenous glutathione (GSH, 1 mM) under drought stress (−0.7 MPa). Mean (±SE) was calculated from three replicates for each treatment. Values in a column with different letters are significantly different at *P* ≤ 0.05 (*) or ≤ 0.01 (**) applying the DMRT.

Source of variation	SOD activity (U min^−1^ mg^−1^ protein)	CAT activity (µmol min^−1^ mg^−1^ protein)	APX activity (µmol min^−1^ mg^−1^ protein)	MDHAR activity (nmol min^−1^ mg^−1^ protein)	DHAR activity (nmol min^−1^ mg^−1^ protein)	GR activity (nmol min^−1^ mg^−1^ protein)	GST activity (nmol min^−1^ mg^−1^ protein)	GPX activity (nmol min^−1^ mg^−1^ protein)
Treatment								
Control	90.53 c	86.67 a	0.656 c	37.71 a	105.42 a	25.67 b	5.65 c	0.064 b
GSH	91.55 c	78.45 a	0.647 c	31.84 b	112.18 a	24.78 b	7.34 c	0.061 b
Drought	112.77 b	63.72 b	0.794 b	25.60 c	80.48 b	26.99 ab	9.60 b	0.068 b
Drought + GSH	125.87 a	75.11 ab	0.947 a	37.90 a	122.84 a	30.81 a	12.29 a	0.092 a
Level of significance	**	**	**	**	**	*	**	**
SE	2.26	2.85	0.018	1.29	4.48	1.40	0.43	0.003
*F* ratio (d.f. = 3,14)	58.08	11.10	66.715	20.44	16.13	3.60	45.48	13.228
Time								
24 h	101.95 b	71.25 b	0.737 b	31.84 b	108.54 a	28.14 a	7.91 b	0.071 b
48 h	108.40 a	80.72 a	0.785 a	34.68 a	101.92 b	25.98 b	9.53 a	0.072 a
Level of significance	**	**	**	**	**	*	**	**
SE	1.59	2.02	0.012	0.91	3.17	0.99	0.30	0.003
*F* ratio (d.f. = 1,14)	8.18	11.00	7.455	4.85	2.18	2.39	14.49	0.022
Treatment × time								
Control × 24 h	91.94 c	79.24 ab	0.624 e	34.35 abc	102.33 bc	25.07 b	4.57 d	0.070 bc
Control × 48 h	89.11 c	94.09 a	0.688 e	41.06 a	108.51 abc	26.26 b	6.72 cd	0.059 c
GSH × 24 h	92.51 c	77.93 abc	0.672 de	31.39 bcd	115.66 ab	25.34 b	7.76 c	0.058 c
GSH × 48 h	90.59 c	78.97 ab	0.622 e	32.29 bcd	108.69 abc	24.21 b	6.93 cd	0.063 c
Drought × 24 h	109.36 b	59.80 c	0.754 cd	24.07 d	80.68 c	28.80 ab	8.39 bc	0.066 c
Drought × 48 h	116.17 b	67.63 bc	0.834 bc	27.13 cd	80.27 c	25.17 b	10.80 b	0.070 bc
Drought + GSH × 24 h	114.00 b	68.03 bc	0.900 ab	37.55 ab	135.47 a	33.36 a	10.90 b	0.090 ab
Drought + GSH × 48 h	137.75 a	82.18 ab	0.994 a	38.25 ab	110.22 ab	28.26 ab	13.67 a	0.095 a
Level of significance	**	**	**	**	**	*	**	**
SE	3.19	4.04	0.026	1.82	6.34	1.98	0.60	0.005
*F* ratio (d.f. = 3,14)	7.47	1.27	3.641	1.17	2.28	0.98	3.75	1.108

### Glyoxalase enzyme activity

Treatments and time, either individually or interactively, significantly affected the activities of the glyoxalase enzymes. Compared with the non-stressed control, Gly I activity did not significantly increase in the drought-affected seedlings after 24 and 48 h. The increase in Gly I activity due to GSH supplementation with drought stress was not significant (compared with drought stress alone) (Table 5). Drought stress notably and significantly increased Gly II activity by 38 and 13 % after 24 and 48 h, respectively, compared with the control seedlings. Exogenous GSH application in the drought treatment increased Gly II activity further after 24 and 48 h (Table 5).

**Table 5.** Activities of Gly I, Gly II and MG content in mung bean seedlings induced by exogenous glutathione (GSH, 1 mM) under drought stress (−0.7 MPa). Mean (±SE) was calculated from three replicates for each treatment. Values in a column with different letters are significantly different at *P* ≤ 0.01 (**) applying the DMRT.

Source of variation	Gly I (µmol min^−1^ mg^−1^ protein)	Gly II (nmol min^−1^ mg^−1^ protein)	MG content (µmol g^−1^ FW)
Treatment			
Control	0.195 c	9.91 c	19.62 d
GSH	0.241 bc	9.32 c	25.78 c
Drought	0.258 ab	12.32 b	39.05 a
Drought + GSH	0.312 a	16.41 a	29.70 b
Level of significance	**	**	**
SE	0.013	0.49	0.83
*F* ratio (d.f. = 3,14)	25.056	43.82	97.00
Time			
24 h	0.242 b	11.19 b	26.48 b
48 h	0.261 a	12.79 a	30.60 a
Level of significance	**	**	**
SE	0.007	0.34	0.58
*F* ratio (d.f. = 1,14)	4.050	10.76	24.88
Treatment × time			
Control × 24 h	0.180 c	8.96 c	18.73 e
Control × 48 h	0.210 bc	10.86 bc	20.50 e
GSH × 24 h	0.235 abc	8.09 c	23.53 de
GSH × 48 h	0.246 abc	10.55 bc	28.03 cd
Drought × 24 h	0.244 abc	12.34 b	35.60 b
Drought × 48 h	0.273 ab	12.31 b	42.50 a
Drought + GSH × 24 h	0.308 a	15.39 a	28.03 cd
Drought + GSH × 48 h	0.316 a	17.43 a	31.37 bc
Level of significance	**	**	**
SE	0.018	0.69	1.17
*F* ratio (d.f. = 3,14)	0.358	1.29	1.71

### Methylglyoxal content

Treatment and time significantly affected MG content and their interactive effect was also significant (at *P* ≤ 0.01). Drought-induced MG toxicity is evident in increased MG content of 90 and 107 %, respectively, after 24 and 48 h of drought stress. Exogenous GSH reduced MG content in the drought-affected seedlings by 21 and 26 % at 24 and 48 h, respectively, compared with drought treatment alone (Table 5).

## Discussion

One of the primary effects of drought stress is reduction in plant growth (Sapeta *et al*. 2013). In the present study, drought stress reduced both DW and leaf area of the mung bean seedlings. However, exogenous GSH restored these growth parameters (but they were not statistically significant in all cases) (Table 2), which is also supported by previous reports. Exogenous GSH supplementation improved the growth of *Arabidopsis thaliana* under drought stress (Chen *et al*. 2012) and improved the growth of *Brassica napus* L. under salt stress (Kattab 2007). Drought reduces tissue water content and leaf RWC (Xu *et al*. 2009; Alam *et al*. 2013). Sustaining leaf RWC under drought stress is considered one of the vital factors in evaluating plant drought stress tolerance (Gonzalez and Gonzalez-Vilar 2001). In the present study, leaf RWC significantly decreased under drought stress (Table 2), which resulted in leaf wilting. Drought also resulted in symptoms of wilting at the whole plant level. However, after exogenous GSH application, RWC was restored or increased significantly, but only at 24 h. Drought stress significantly reduced leaf succulence and it was restored by exogenous GSH application with a significant value for 24 h. Leaf succulence is considered the capacity to store water per unit leaf area and a plant is more drought resistant if it possesses a high water storage capacity (Larcher 1986). Proline, which is an osmoregulator, increased significantly (Table 2) in the exogenous GSH-treated drought-affected seedlings, which is one reason for increasing water status parameters such as RWC and leaf succulence. It is surmised that exogenous GSH through increasing the Pro level maintains osmotic potential, which might help to maintain plant water content. Adequate water content is also essential for maintaining plant growth and physiology. Reduced photosynthetic pigment content including chl *a*, chl *b*, total chl and carotenoid under drought stress has been reported in several plant species, such as *Avena* sp., *Triticum* sp. and *Gossypium* sp. (Parida *et al*. 2007; Chakraborty and Pradhan 2012; Pandey *et al*. 2012). The mung bean seedlings exposed to drought stress in this study showed similar reductions in chl *a*, chl *b* and total chl (*a* + *b*) content (Table 2). The reasons for reduced photosynthetic pigment might be changes in chloroplast structure or inhibited biosynthesis of chl or its precursors (Plesnicár *et al*. 1997). Exogenous GSH prevented the reduction of chl *a* and total chl content under drought stress. In a previous study, Alam *et al*. (2013) showed that in *Brassica juncea* L. under drought stress, a higher endogenous GSH level was associated with higher chl *a*, chl *b* and total chl content. Glutathione is able to prevent or retard chl degradation resulting from oxidative stress. Glutathione through reducing oxidative stress may protect chl biosynthesis enzymes, which might be related to higher chl content (Kattab 2007). Increased Pro level is an indicator of drought-induced stress (Rampino *et al*. 2006; Alam *et al*. 2013). Our results also show significant increases in Pro level, and even with exogenous GSH application with drought, Pro was still maintained at an elevated level (Table 2). Proline is a compatible solute and it plays crucial roles in reducing stress-induced cellular acidification and maintaining osmoregulation, and acts as an osmoprotectant (Hasegawa *et al*. 2000), which can prevent water loss or reduction of leaf RWC (Ahmed *et al*. 2011). An elevated Pro level correlates with higher RWC and leaf succulence (Table 2), which were considered as water status parameters in the present study. Proline stabilizes macromolecules (Khedr *et al*. 2003; Ashraf and Foolad 2007); it can act as a hydroxyl radical (OH^·^) and singlet oxygen scavenger, a nitrogen and carbon source needed in stress recovery, and a component of stress signal transduction pathways (Aziz *et al*. 1998; Khedr *et al*. 2003). Higher Pro levels even after exogenous GSH application might also have a beneficial role in reducing oxidative damage (Table 1).

Drought can damage cell membranes, chloroplast membranes or other sub-organelles; disturbs the mechanism of photosynthesis (Nayyar and Gupta 2006); can inactivate enzymes of the Calvin cycle; reduces the efficiency of the carboxylation reaction and CO_2_ fixation by RuBisCO (Ribulose-1,5-bisphosphate carboxylase/oxygenase) and may increase photorespiration, which might render overproduction of ROS beyond plant scavenging capacity (Hoekstra *et al*. 2001; Monakhova and Chernyad'ev 2002). Thus, drought exposes plants to severe oxidative stress. In the present study, histochemical detection shows clear indications of oxidative stress as brown spots of H_2_O_2_ and dark blue spots of ${{\text{O}}_{2}}^{\cdot -}$ in mung bean leaves under drought stress (Figs 1B and 2B). Generation of ${{\text{O}}_{2}}^{\cdot -}$ is induced in drought-affected plant cells through impaired electron transport in the chloroplasts and this ${{\text{O}}_{2}}^{\cdot -}$ can be converted into H_2_O_2_, which triggers various symptoms in water-stressed plants (Price *et al*. 1989; Wang *et al*. 2005). Similar visual identification of H_2_O_2_ and ${{\text{O}}_{2}}^{\cdot -}$ was reported previously as brown patches and dark blue spots, respectively, in drought-affected plants (Jiang *et al*. 2013; Pyngrope *et al*. 2013). Drought caused a noticeable increase in the ${{\text{O}}_{2}}^{\cdot -}$ generation rate and H_2_O_2_ level with a marked increase in lipid peroxidation (indicated by higher MDA level) after 24 h. After 48 h of drought stress, the mung bean seedlings suffered more severe oxidative stress (Table 1), which is in line with previous studies (Bian and Jiang 2009; Duan *et al*. 2009; Wang *et al*. 2012). Exogenous GSH application with drought stress significantly reduced oxidative stress (reduced leaf spots and contents of ROS and lipid peroxidation) in the mung bean seedlings, which shows the beneficial role of exogenous GSH, and the reduction in oxidative stress was higher at 24 h compared with 48 h (Table 1; Figs 1 and 2). Glutathione, as a potential scavenger of ^1^O_2_, H_2_O_2_ and OH^·^, counteracts the inhibitory effects of ROS-induced oxidative stress and maintains the normal reduced state of cells (Larson 1988; Briviba *et al*. 1997; Noctor and Foyer 1998). Reduction of oxidative stress by exogenous GSH under drought stress in the present study is also supported by Chen *et al*. (2010), who demonstrated that GSH reduced the brown spots of H_2_O_2_ and dark blue spots of ${{\text{O}}_{2}}^{\cdot -}$ in cadmium-stressed barley leaves. Moreover, adding exogenous GSH enhanced other non-enzymatic (Table 3) and enzymatic components (Table 4) of the antioxidant system, which might work to a great extent to prevent oxidative damage in plants.

Lipoxygenase activity is responsible for ROS generation in various ways. Singlet oxygen and superoxide anions can be formed during LOX-catalysed fatty acid oxidation (Lynch and Thompson 1984). Increased LOX activity has been shown to cause increased lipid peroxidation under stress conditions including drought (Aziz and Larher 1998; Sánchez-Rodríguez *et al*. 2010). Similar increased LOX activity was found to correlate with the oxidative damage in the present study (Table 1). In contrast, lower LOX activity (Table 1) in the exogenous GSH-supplemented drought-stressed seedlings was associated with lower MDA and H_2_O_2_ levels (Table 1). Similar reduced oxidative stress with reduced LOX activity was observed in *Solanum lycopersicum* (Sánchez-Rodríguez *et al*. 2010).

Components of the antioxidant system, either non-enzymatic or enzymatic, help to decrease oxidative damage and improve drought tolerance and resistance in plants (Sharma and Dubey 2005; Jaleel *et al*. 2009). Ascorbate is an efficient primary scavenger of ROS (Foyer and Noctor 2011). In this experiment, drought stress significantly reduced the AsA content (Table 3), which might be one reason for oxidative stress (Table 1). Later on, application of exogenous GSH improved the AsA level in drought-affected seedlings, which is one reason for reduced oxidative stress (induced by drought). After taking part in the ROS scavenging process, AsA is oxidized and afterward can be recycled back to its reduced AsA form by the activities of MDHAR and DHAR (Foyer and Noctor 2011; Hasanuzzaman *et al*. 2012). Drought stress in this experiment reduced the activities of MDHAR and DHAR (Table 4), which reduce the AsA level (Table 3). Enhanced APX activity might also reduce AsA content as it is responsible for higher DHA generation. Higher APX activity is also responsible for direct scavenging of H_2_O_2_ to H_2_O (Foyer and Noctor 2011; Hasanuzzaman *et al*. 2012). The results of this study showed that drought caused higher APX activity (Table 4) compared with the control, which agrees with previous findings where drought caused higher APX activity and reduced MDHAR and DHAR activities with reduced AsA level in *B. napus* (Hasanuzzaman and Fujita 2011; Alam *et al*. 2013). However, exogenous GSH application enhanced MDHAR activity for both 24 and 48 h and DHAR activity for 24 h only. Enhanced MDHAR and DHAR activity (Table 4) induced by exogenous GSH application was able to effectively recycle the potent antioxidant AsA to scavenge ROS. It has also been reported that GSH plays a key role in regenerating the water soluble antioxidant AsA, via the ASH–GSH cycle (Foyer and Halliwell 1976), which might also contribute to increasing the AsA level of exogenous GSH-added drought treatment. The endogenous GSH level of the mung bean seedlings increased significantly for both durations of drought stress (Table 3), which is in line with previous findings (Hasanuzzaman and Fujita 2011; Alam *et al*. 2013). Moreover, exogenous GSH application increased the endogenous GSH levels (Table 3), which might be a preventive mechanism, as a higher GSH level was shown to effectively quench free radicals and reduce oxidative damage (Lu 2009). After scavenging ROS, GSH is oxidized to GSSG (Asada 1994). In this study, GSSG content was very high under drought stress, compared with the control (Table 3). Although the reduction of GSSG in the 24 h treatment was not statistically significant, its reduction due to exogenous GSH application with drought might be an indicator of reduced oxidative stress. Glutathione disulfide can be catalysed back to GSH by the enzyme GR (Hasanuzzaman *et al*. 2012). However, in the present study, GR activity (Table 4) slightly increased after exogenous GSH application; thus, it might be involved in recycling and increasing endogenous GSH (Table 3). Moreover, the higher GSH level of the mung bean seedlings might be contributed to by the GSH biosynthesis process.

The GSH/GSSG ratio indicates the intracellular redox potential and plays an important role in the signalling process of various stresses (Forman *et al*. 2009; Lu 2009). Plants with a higher GSH/GSSG ratio possess higher stress-tolerance characteristics. The GSH/GSSG ratio was greatly reduced by drought stress. Exogenous GSH application with drought reversed this condition by increasing the GSH/GSSG ratio (Table 3). Previous research explored whether reduction in MDA and H_2_O_2_ levels correlated to increased GSH content, and the GSH/GSSG ratio with enhanced activities of the antioxidant enzymes (Hasanuzzaman and Fujita 2011).

Superoxide dismutase provides frontline defence against ROS by removing ${{\text{O}}_{2}}^{\cdot -}$ (Hasanuzzaman *et al*. 2012). A significant increase in SOD activity was observed in drought-affected mung bean seedlings after 24 and 48 h (Table 4). This increase defends against ROS, which was generated due to drought stress and this corroborates the findings of a previous study (Zhang and Kirkham 1994). Exogenous GSH addition with drought maintained the same SOD activity in the 24 h treatment and significantly increased it in the 48 h treatment, compared with the drought treatment alone. Catalase is a tetrameric heme-containing enzyme that uses H_2_O_2_ as a substrate and converts it to H_2_O and O_2_ (Sánchez-Casas and Klessig 1994). Catalase activity in the mung bean seedlings decreased after drought exposure (Table 4) and could not be significantly increased with exogenous GSH. Induction of GST activity under abiotic stress conditions has been considered an important factor in stress-tolerance development in plants (Dixon *et al*. 2010; Hasanuzzaman *et al*. 2012). Compared with the control, the GST activity of the drought-stressed seedlings increased, and GSH application further increased GST activity in the drought-affected seedlings with a significant increase in the 48 h treatment (Table 4). Glutathione acts as a substrate for GPX and helps to convert toxic ROS into a non-toxic state (Noctor *et al*. 2002; Hasanuzzaman *et al*. 2012). Glutathione is used by GPX during scavenging of H_2_O_2_ and lipid hydroperoxides and therefore protects plant cells from oxidative stress (Herbette *et al*. 2002). Thus, increased endogenous GSH (Table 3) together with enhanced GPX activity by exogenous GSH (Table 4) might have a beneficial role in scavenging ROS under drought stress, which is in line with a previous study (Hasanuzzaman and Fujita 2011).

From the above discussion, it is evident that exogenous GSH was able to reduce drought-induced oxidative damage in drought-affected mung bean seedlings. Decreasing drought-induced oxidative damage was achieved by increasing the content of non-enzymatic antioxidants including endogenous GSH and AsA levels, and increasing the activities of antioxidant enzymes by exogenous GSH to scavenge ROS.

Abiotic stresses result in significant increases in MG content that can greatly amplify ROS production and can thus be responsible for oxidative and cytotoxic stress (Yadav *et al*. 2005*b*; Singla-Pareek *et al*. 2006; Turóczy *et al*. 2011). In the glyoxalase system, using GSH as substrate, the enzymes Gly I and Gly II effectively detoxify MG, which is desirable under drought stress (Hoque *et al*. 2008). The rise in MG content due to drought stress is evident in the mung bean seedlings in the present study (Table 5). The MG levels were reduced by exogenous GSH application with drought stress (Table 5) and this result indicates the vital role of GSH in MG detoxification (Yadav *et al*. 2005*b*, *c*). The increase in Gly II activity was significant in the GSH-supplemented drought-affected seedlings and there was no significant increase in Gly I activity, compared with the drought-affected plants (Table 5). Increased Gly I and Gly II activities also contributed to MG detoxification with increased endogenous GSH levels (at both 24 and 48 h; Table 3), which corroborates previous research findings (Yadav *et al*. 2005*b*, *c*; Hasanuzzaman and Fujita 2011; Nahar *et al*. 2015*a*). Exogenous GSH application significantly increased the endogenous GSH level in the drought-affected seedlings, and may act as a substrate for enzymes of the glyoxalase system to detoxify the MG generated as a result of drought stress. Moreover, exogenous GSH enhanced the activities of the glyoxalase system. Thus, the exogenous GSH-induced enhanced glyoxalase system including the glyoxalase enzymes and GSH content contribute to coping with drought stress.

## Conclusions

The results of the present study demonstrate that exogenous GSH application improved mung bean seedling growth and physiological performance under drought stress. Several mechanisms were involved in exogenous GSH-induced alleviation of drought damage in the mung bean seedlings. Maintaining water status is the prerequisite for conferring drought tolerance. Glutathione maintained osmotic balance by regulating Pro content and thus improved the water status of plant cells, maintaining adequate tissue water content. Glutathione effectively eliminated drought-induced oxidative damage not only by acting as an efficient antioxidant but also improving other components of the antioxidant system. Glutathione effectively detoxified and reduced MG by improving the glyoxalase system. Glutathione also improved the physiological condition of the mung bean seedlings under drought stress. Exogenous GSH application was more effective against 24 h of drought stress, compared with 48 h of drought stress. The role of endogenous GSH in antioxidant defence, cellular redox regulation, stress signalling and abiotic stresses including drought tolerance has been well studied (Hasanuzzaman and Fujita 2011; Hasanuzzaman *et al*. 2011). However, the role of exogenous GSH application as a protectant against different abiotic stresses has not been adequately studied, especially its role under drought stress. Therefore, extensive studies are needed to explore the role of exogenous GSH in plants under drought stress.

## Sources of Funding

We are grateful to the Ministry of Education, Culture, Sports, Science and Technology (MEXT), Japan for financial support.

## Contributions by the Authors

Among the authors, K.N. has designed and implemented the experiment, prepared the manuscript. M.H. had substantial contributions to the conception and design, and/or acquisition of data, and/or analysis and interpretation of data. He also participated in drafting the article or revising it critically for important intellectual content. M.M.A. actively participated in executing experiment. M.F. as a corresponding author had vital contribution in designing experiment, reviewing critically for important intellectual content and giving final approval of the version to be submitted.

## Conflict of Interest Statement

None declared.
